# Mothers' Knowledge and Adherence to Who Breastfeeding Recommendations: A Cross-Sectional Study in Greece

**DOI:** 10.34763/jmotherandchild.20252901.d-25-00013

**Published:** 2025-08-16

**Authors:** Ermioni Palaska, Eirini Orovou, Maria Iliadou, Kleanthi Gourounti, Paraskevi Giaxi, Anastasia Bothou

**Affiliations:** Research Laboratory of Midwifery Care During Antenatal and Postnatal Period-Breastfeeding, Department of Midwifery, School of Health & Care Sciences, University of West Attica, Athens, Greece; Department of Midwifery, University of Western Macedonia, Ptolemaida, Greece

**Keywords:** breastfeeding, exclusive breastfeeding, infant feeding

## Abstract

**Background:**

Breastfeeding is the ideal nutrition for the first half of an infant's life. It contains the right nutrients for the physical and mental development of the infant. In general, however, fewer than 50% of infants under six months are exclusively breastfed globally. This study aims to determine if Greece's exclusive breastfeeding rates comply with WHO and UNICEF recommendations and to explore the variables that affect the lengthening of exclusive breastfeeding.

**Material and methods:**

This cross-sectional study was conducted in four municipalities of South Athens, Greece from July 2018 to June 2019 in day care centers. The sample of study consisted of 674 mother-child couples. The study was approved by the Department of Nursing at the University of the Peloponnese, as well as the municipal day care centers. The mothers were informed about the purpose of the study.

**Results:**

236 (35.01%) women in the sample breastfed exclusively for six months or more, of which 106 (44.91%) women continued breastfeeding for one year, and 22 (9.32%) women until two years. Exclusive breastfeeding, satisfaction with the process, gestational diabetes, excess and normal maternal weight at the time of the study are statistically significant factors for increasing the duration of exclusive breastfeeding.

**Conclusions:**

Exclusive breastfeeding rates in Greece fall short of WHO and UNICEF targets, with only 35.01% of mothers breastfeeding exclusively for six months.

## Introduction

Breastfeeding is the biological norm for feeding infants. When used properly, it offers a multitude of health benefits for both mother and child. [1,2,3]. The World Health Organization (WHO) advises that infants should start breastfeeding within the first hour of life and continue exclusively for the first six months. After six months, breastfeeding should be complemented by appropriate and safe complementary foods, continuing until the child is at least two years old or older [3]. It has been observed that breastfeeding expedites the mother's process of returning to her prepregnancy weight and the uterus's shrinkage to its prepregnancy size. Moreover, breastfeeding has been demonstrated to lower the risk of breast cancer (particularly in cases when a woman has been breastfeeding for > 12 months) [4,5,6], ovarian cancer, dyslipidemia, and type 1 and type 2 diabetes mellitus. In addition, breastfeeding offers the infant numerous advantages because it supplies all the nutrients it needs in the right amounts and qualities depending on its developmental stage. It also shields the infant from infections like ear, gastrointestinal, and respiratory infections and helps prevent food allergies from developing in the future [7].

According to WHO and UNICEF, many infants and children do not receive optimal nutrition [8]. Several studies confirm that breastfeeding rates worldwide remain below the levels needed to protect maternal and child health [8,9,10,11,12]. For example, Victora et al. (2016) found that only 36% of infants aged 0–6 months were exclusively breastfed between 2007 and 2014 [9], while WHO data from 2017 show that just 41% of infants under six months were exclusively breastfed [11-12]. The WHO collective targets for global rates in 2030 are 70% of women to breastfeed their newborn in the first hour, 70% to breastfeed exclusively for six months of which 80% of women to continue breastfeeding for one year, and 60% for two years [13,14].

The present study aims to investigate the rates of breastfeeding in Greece according to WHO and UNICEF guidelines.

## Material and methods

This is a cross-sectional study that was carried out in four day care centers of South Athens, Greece, from February 22, 2018, to June 30, 2019. The sample of study consisted of 674 mother-child couples. The mothers were informed about the purpose of the study by the researcher. Also, they were informed about the confidentiality of the data and the anonymity of the study. The mothers also filled out the consent form for participation in the research. The questionnaires were completed by the mothers, who, after one week, returned them to the researcher.

### Study population: Selection and exclusion criteria

The study was conducted in the kindergartens of southern Athens, Greece. The sample consisted of 674 women with children aged 2 to 5. Mothers who did not speak Greek were excluded from the sample collection.

### Measure

In the present cross-sectional study, a questionnaire on breastfeeding was used. The reason for choosing this method is that it is more suitable for gathering a large number of participants and because it produces more reliable results. The questionnaire concerned pregnancy, childbirth, and breastfeeding. In addition, the questionnaire was weighted. In this study, content validity and test-retest procedures were used to weigh the questionnaire to check its reliability. The questionnaire was sent to experts informing them about the objectives of the research and the areas it refers to (pregnancy, childbirth, and breastfeeding). This was followed by revisions until the version used in this study was finalised. To check for reliability, the questionnaire was given to the respondents twice, two months apart. The score of the first measurement was then correlated with the score of the second measurement for the pregnancy–birth unit and for breastfeeding separately. The nonparametric Spearman's rho test was used for correlation. For the pregnancy-childbirth questions, a statistically significant positive correlation emerged between the first and second measurement (rho = .751, *p* < .001). Still, for the breastfeeding questions, a statistically significant positive correlation emerged between the first and the second measurement (rho = .900, *p* < .001). Based on the results, the questionnaire is considered reliable, as a high level of positive correlation was found between the two measurements.

### Ethical approval

During this study, all the fundamental ethical principles governing the conduct of a research study were observed. In particular: (a) The questionnaires were completed by the participants with their consent after being fully informed about the study and its contribution to the scientific and social community. (b) The material collected was kept confidential and used only for the research. (c) The study was approved by the Department of Nursing at the University of the Peloponnese (N. 1341/22/2/2018 as well as by the four municipal day care centers (N. 19609/05-09-2018, N. 2600/11-09-2018, N. 571/13-9-2018, N. 521/22-02-2019).

### Statistical analysis

The results were processed using the statistical package SPSS (version 22.0, IBM Corp., Armonk, NY, USA). To address potential confounding variables, multiple logistic regression analysis was also performed. A *p*-value of less than 0.05 was designated as the statistically significant level.

## Results

The average age of mothers was 36.91 years (SD = 4.51), with the youngest mother being 24 years old and the oldest 56 years old. The average age at pregnancy was 33.93 years (SD = 4.52), with the youngest mother at pregnancy being 20 years old and the oldest 52 years old.

Regarding health and lifestyle during pregnancy, 80 women (11.9%) reported developing gestational diabetes, while 591 women (87.7%) did not. Furthermore, 71 women (10.5%) reported smoking during pregnancy, while 602 women (89.4%) did not. Additionally, 46 women (6.8%) reported drinking alcohol during pregnancy, with 627 women (93.1%) abstaining from alcohol. In terms of maternal weight, 486 women (72.1%) had a normal weight, 85 women (12.6%) were overweight, 50 women (7.4%) were underweight, and 47 women (7%) were obese (Table 1). These findings highlight a relatively high average maternal age, which reflects a trend in delayed pregnancies. While most women appeared to be in good health, with high percentages not engaging in smoking, drinking alcohol, or developing gestational diabetes, a significant proportion of women were either overweight or obese, with around 20% of the participants falling into these categories.

The type of delivery was reported by the 674 women: 244 (36.2%) women gave birth by normal delivery, 26 (3.86%) women gave birth by vacuum extraction delivery, and 404 (59.94%) women by cesarean section. Epidural anaesthesia was performed in 561 (83.2%) women, of which 291 (51.9%) women reported that epidural anaesthesia was their personal choice. Breastfeeding was also reported by the women: 650 (96.54%) women reported that they breastfed the newborn, while 24 (3.56%) women reported that they did not breastfeed; 629 (93.3%) women reported breastfeeding the first hour after birth of the newborn, while 44 (6.5%) women did not breastfeed (Table 1).

**Figure 1.**
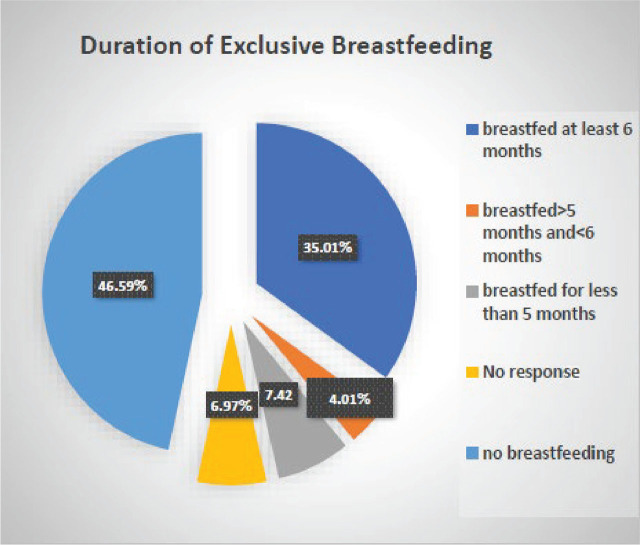
Duration of exclusive breastfeeding.

The length of breastfeeding was also recorded: 236 (35.01%) women breastfed exclusively for at least six months; 27 (4, 01 %) women breastfed > 5 months and < 6 months; and 50 (7.42%) women breastfed < 5 months. Of the 236 women who breastfed at least 6 months, 106 (44.91%) continued breastfeeding for one year, and 22 women (9.32%) continued for two years (Fig. 1, Table 1).

A total of 246 women (36.5%) reported that their newborn was immediately removed after birth, while 387 women (57.4%) did not experience this, and 40 women (5.9%) did not provide a response. The average time taken to transfer the neonate to the room for women whose babies were removed after delivery was 6.28 hours (SD = 8.77), with the shortest time being 1 hour and the longest 48 hours. Regarding infant feeding in the first few days, 330 women (49%) reported that their newborns were given formula milk, while 332 women (49.3%) did not use formula milk. Among the 330 women who indicated their infants received formula milk, 234 (70.9%) said they were aware of it, 37 (11.21%) were not aware, and 59 (17.87%) did not respond. Of the 27 women who answered why formula milk was given, the reasons provided included jaundice, the baby being underweight, insufficient milk supply, the baby being in an incubator, a health problem, or the baby needing to stay in the hospital.

**Table 1.** Demographic and clinical characteristics of all participants.

**Data**	**Frequency**	**Percentage**
**Gestational diabetes**	N	%
Yes	80	11.9
No	591	87.7
Missing data	3	0.4
**Smoking during pregnancy**		
Yes	71	10.5
No	602	89.4
Missing data	1	0.1
**Alcohol consumption during pregnancy**		
Yes	46	6.8
No	627	93.1
Missing data	1	0.1
**BMI**		
Underweight	50	7.4
Normal weight	486	72.1
Overweight	85	12.6
Obese	47	7.0
Missing data	6	0.9
**Delivery**		
Normal delivery	244	36.2
Cesarean section	404	59.94
Vacuum extraction delivery	26	3.86
**Administration of epidural anaesthesia**		
Yes	561	83.2
No	99	14.7
Missing data	14	2.1
**Personal choice: administration of epidural anaesthesia**		
Yes	291	51.9
No	24	4.3
Missing data	246	43.9
**Breastfeeding the first hour after delivery**		
Yes	629	93.3
No	44	6.5
Missing data	1	0.1
**Breastfeeding**		
Yes	650	96.54
No	24	3.56
Missing data	0	
**Duration of exclusive breastfeeding**		
Exclusive breastfeeding at least 6 months	236	35.01
Exclusive breastfeeding > 5 months and < 6 months	27	4.01
Exclusive breastfeeding < 5 months	50	7.42
No breastfeeding	314	46. 59
Missing data	47	6.97
**Exclusive breastfeeding at least 6 months: women who continued breastfeeding**		
for 1 year	106	44.91
for 2 years	22	9.32
**Questions about breastfeeding and milk sufficiency Has anyone told you that you don't have enough milk?**		
Yes	135	20.3
No	529	78.49
No response	10	1.48
**Did anyone tell you that your milk is too thin?**		
Yes	62	9.20
No	600	89.02
No response	12	1.78
**Has anyone told you that your nipples are not good?**		
Yes	91	13.5
No	571	84.72
No response	12	1.78

**Figure 2.**
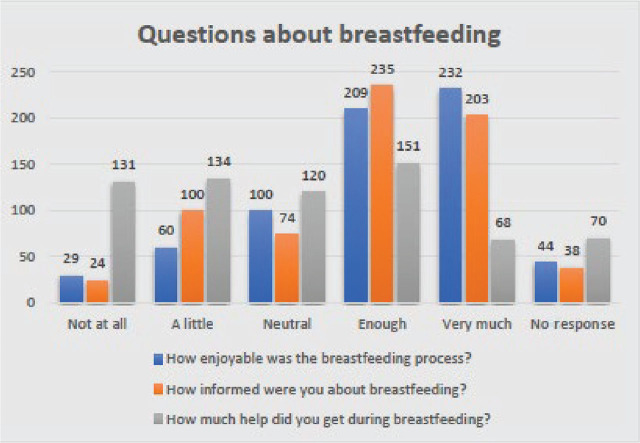
Questions about breastfeeding.

**Table 2.** Multiple linear model of breastfeeding duration prediction.

**Model**	**Nonstandardized Coefficients**		**Standardized Coefficients**	**t**	**Sig.**	**Collinearity Statistics**	
	**B**	**Std. Error**	**Beta**			**Tolerance**	**VIF**
(Constant)	−.774	4.310		−.180	.858		
Exclusive breastfeeding (YES = 1, 2 = NO)	−7.004	.910	−.363	−7.695	.000	.936	1.068
Degree of pleasure from breastfeeding	1.898	.407	.223	4.666	.000	.913	1.095
Smoking (YES = 1, 2 = NO)	4.127	1.498	.126	2.755	.006	.993	1.007
Overweight mother now (versus underweight)	4.355	1.375	.155	3.166	.002	.873	1.146
Normal maternal weight now vs. underweight	1.951	.928	.103	2.103	.036	.868	1.152
Gestational diabetes (YES = 1, 2 = NO)	2.644	1.303	.093	2.030	.043	.997	1.003

*Note:* B = nonstandardized beta; P = *p* value; t = *t* test; VIF = variance inflation factor.

The women recorded the reasons for not breastfeeding: 135 (20.03%) women reported that they were told they did not have enough milk, 91 (13.5%) women that their nipples were not good, and 62 (9.2%) women that their milk was thin (Table 1).

They also reported on the information they were given about breastfeeding: 592 (87.3%) women were informed about breastfeeding, while 66 (9.79%) women were not informed, and 16 (2.37%) women did not respond. Regarding the percentage of 592 women who were informed about breastfeeding, 245 (41.4%) women said they were informed by the midwife, 252 (42.6%) women by staff, 100 (16.9%) women by the doctor, 6 (1%) women by the Internet, and 47 (7.9%) women by other sources (books, friends, seminars, relatives, first child, etc.).

The ease of breastfeeding was elicited from the woman: 441 (65.43%) women reported that the breastfeeding process was easy enough and very pleasant; 100 (14.84%) women had a neutral attitude; 438 (64.99%) women reported that they were enough and very informed about breastfeeding; 74 (10.98%) women had a neutral attitude; 219 (32.49%) women reported that they had a lot of help during breastfeeding; 120 (17.80%) women had a neutral attitude; and 131 (19.44%) of the women did not report on the availability of help at all (Fig. 2).

The women expressed their feelings about breastfeeding: 129 (19.14%) women reported that they would like to change something during breastfeeding, while 356 (52.82%) women did not want any change. Of the women who wished to change something, 20 (15.50%) women would like to be calmer, 19 (14.73%) women would like to be better informed, 11 (8.53%) women would like to breastfeed normally and not with the use of a breast pump, 11 (8.53%) women would like to breastfeed more, and the remaining 58 (44.96%) women reported 26 different reasons.

Based on the multiple linear model, the dependent variable was the breastfeeding interval in months while the independent variables were normal delivery, giving foreign milk in the first few days, exclusive breastfeeding, third-party report of not having enough milk or having thin milk, third-party report of not having good nipples, information about breastfeeding, degree of enjoyment of breastfeeding, degree of information about breastfeeding, assistance during breastfeeding, and removal of the newborn after delivery. The stepwise method was used to find the optimal model (this method starts by introducing variables into the model until there is no improvement, at which point it stops). The final model was found to be statistically significant, F (6. 345) = 21.996, *p* < .01, R square = .275. The model had no problems with multilinearity (VIF < 10) and autocorrelation (Durbin Watson = 1.966, acceptable values 1–3).

Statistically significant predictor variables for the duration of exclusive breastfeeding include exclusive breastfeeding itself (b = 7.004, *p* < .01), the degree of pleasure from breastfeeding (b = 1.898, *p* < .01), gestational diabetes (b = 2.644, *p* < .05), being overweight (b = 4.355, *p* < .01), and having a normal weight (b = 1.951, *p* < .01). Women who exclusively breastfeed are able to breastfeed 7.004 months longer than those who do not. Women without gestational diabetes breastfeed 2.644 months longer than those with gestational diabetes. Additionally, for every one-unit increase in the degree of pleasure from breastfeeding, the duration of breastfeeding increases by 1.898 months. Overweight women (BMI > 25) breastfeed 4.355 months longer compared to underweight women (BMI < 20), while women with a normal weight (BMI between 20 and 25) breastfeed 1.951 months longer than underweight women (Table 2).

## Discussion

### Main findings

The rates of breastfeeding in Greece are low, and in particular, the rates of exclusive breastfeeding amount to less than 1% at the end of the sixth month, based on the National Breastfeeding Studies.

The study by Theofilogiannakou et al. (2006) showed that the rate of exclusive breastfeeding on the last day of hospital stay reached 85%, while at 40 days after delivery it decreased to 35%, and after 6 months to 12% [15].

According to the national study of breastfeeding rates, carried out in 2009 in Greece, initiation of breastfeeding is observed at very high rates (87%); however, exclusive breastfeeding immediately after birth is very low (41%). Regarding the continuation of breastfeeding, these percentages decrease after the first month and reach 0.8% of exclusive breastfeeding in the first six months of life [16].

In the national study conducted in 2017, the percentage of women who breastfed on the first day of life was 94%. The percentage of infants who breastfed exclusively in the first 24 hours was found to be 66%. Then, the reduction in rates was rapid, ending in almost zero rates (0.8%) at the end of the sixth month [17].

### Comparison with previous literature

According to WHO, approximately 44% of infants aged 0–6 months are exclusively breastfed [3]. In the current study, an improvement is observed from earlier rates of exclusive breastfeeding in Greece because the rate of exclusive breastfeeding was ≥ 6 months for 236 (35.01%) of the women. Of the 236 (35.01%) women who breastfed for at least 6 months, 106 (44.91%) women continued breastfeeding for one year and 22 (9.32%) women breastfed for two years. In the current study, we observe that the percentage of exclusive breastfeeding in Greece has increased, but it is less than 70% for the first semester recommended by the WHO for the optimal development of the child. Also, the research by Tiga et al. (2021), in five maternity hospitals in Athens in a sample of 847 women for the year 2020 showed an increased rate of breastfeeding in the first semester without the administration of foreign milk, amounting to 30.7% [18].

### Factors affecting breastfeeding

The problems that lead to the failure of exclusive breastfeeding for six months are the administration of foreign milk to the newborn and the infant, the lack of information about breastfeeding, the delay in starting breastfeeding after birth, the type of delivery, the administration of general anaesthesia, insufficient milk production, maternal obesity, smoking, and lack of information. [11,12,19].

In the present research, no statistically significant relationship was found for the effect of the type of delivery, the administration of foreign milk, the administration of general anaesthesia, information about breastfeeding, and insufficient milk production in the maintenance of exclusive breastfeeding. On the contrary, exclusive breastfeeding, and women's satisfaction with breastfeeding, are a statistically significant factor for the duration of exclusive breastfeeding.

### Strengths and limitations

One limitation of the present study is that participation was limited to Greek-speaking mothers of children aged 2 to 5 years, which may affect the generalizability of the results.

The rates of breastfeeding in the first semester, the first year, and the second year are lower than the recommended rates of the WHO but are more than those found in previous national studies in Greece. Because there are no official data in Greece on the rates of breastfeeding, it is considered important for the breastfeeding committee to record the rate of exclusive breastfeeding in the first semester, and the continuation of breastfeeding in the first year and in the second year so that there are objective data.

## Conclusion

Breastfeeding rates in Greece remain below WHO and UNICEF targets, although recent data suggest improvement. Exclusive breastfeeding and maternal satisfaction were identified as key factors influencing its duration. Promoting and supporting breastfeeding should be a public health priority, and further research is needed to better understand and enhance exclusive breastfeeding practices during the first six months of life.
